# IL-36, IL-37, and IL-38 Cytokines in Skin and Joint Inflammation: A Comprehensive Review of Their Therapeutic Potential

**DOI:** 10.3390/ijms20061257

**Published:** 2019-03-13

**Authors:** Marie-Astrid Boutet, Alessandra Nerviani, Costantino Pitzalis

**Affiliations:** Centre for Experimental Medicine & Rheumatology, William Harvey Research Institute and Barts and The London School of Medicine and Dentistry, Queen Mary University of London, London E1 4NS, UK; m.a.boutet@qmul.ac.uk (M.-A.B.); a.nerviani@qmul.ac.uk (A.N.)

**Keywords:** psoriasis, psoriatic arthritis, rheumatoid arthritis, interleukin-36, interleukine-1, interleukin-37, interleukin-38, TLR

## Abstract

The interleukin (IL)-1 family of cytokines is composed of 11 members, including the most recently discovered IL-36α, β, γ, IL-37, and IL-38. Similar to IL-1, IL-36 cytokines are initiators and amplifiers of inflammation, whereas both IL-37 and IL-38 display anti-inflammatory activities. A few studies have outlined the role played by these cytokines in several inflammatory diseases. For instance, IL-36 agonists seem to be relevant for the pathogenesis of skin psoriasis whereas, despite being expressed within the synovial tissue, their silencing or overexpression do not critically influence the course of arthritis in mice. In this review, we will focus on the state of the art of the molecular features and biological roles of IL-36, IL-37, and IL-38 in representative skin- and joint-related inflammatory diseases, namely psoriasis, rheumatoid arthritis, and psoriatic arthritis. We will then offer an overview of the therapeutic potential of targeting the IL-36 axis in these diseases, either by blocking the proinflammatory agonists or enhancing the physiologic inhibitory feedback on the inflammation mediated by the antagonists IL-37 and IL-38.

## 1. Introduction

The interleukin (IL)-1 family of cytokines includes seven agonists (IL-1α, IL-1β, IL-18, IL-33, IL-36α, IL-36β, and IL-36γ) and four antagonists (IL-1 receptor antagonists (Ra), IL-36Ra, IL-37, and IL-38). These cytokines play a significant role in both the innate and acquired immunity by either promoting the resolution of infection or favoring inflammation through their binding to one of the ten receptors and coreceptors of the IL-1R family. In homeostatic conditions, the expression and activity of these cytokines and receptors are finely regulated; in contrast, an unrestrained expression or uncontrolled activation can initiate or enhance a pathologic inflammatory response.

IL-36, IL-37, and IL-38 are the most recently discovered members of the IL-1 family. Their encoding genes, firstly cloned in 2001, are located on chromosome 2 [1]. Although their molecular mechanisms have yet to be fully elucidated, several studies have already emphasized the potential therapeutic value of targeting the IL-36 axis during skin and joint inflammation.

In this review, we will focus on three representative inflammatory diseases of skin and joints: psoriasis, psoriatic arthritis (PsA), and rheumatoid arthritis (RA). Psoriasis is an autoimmune disease affecting ~1% of the population worldwide [2] and characterized by the formation of inflamed, red, and scaly patches on the skin. Up to 30% of patients with skin psoriasis develop a chronic seronegative spondyloarthropathy named PsA and clinically defined by the presence of spondylitis, enthesitis, or peripheral arthritis [3]. The diagnosis of PsA is sometimes laborious because of the huge variety of the presenting manifestations. It relies on the classification criteria published by the CASPAR (ClASsification criteria for Psoriatic ARthritis) group, which include (i) evidence of psoriasis, (ii) psoriatic nail dystrophy, (iii) negative tests for RA, (iv) dactylitis, and (v) radiographic evidence of juxta-articular new bone formation [4]. Although psoriasis and PsA share common features such as genetic susceptibility, comorbidities, or certain pathogenic immunologic pathways, several important tissue-specific differences exist, as highlighted in a recent publication from our group [5]. RA is the most common chronic autoimmune joint disease, impacting ~0.3% of the population worldwide [6]. It is characterized by the inflammatory hyperplasia of the synovial membrane of diarthrodial joints and the subsequent cartilage destruction and bone erosions. The progressive damage of the articular structures causes disabilities and impairs patients’ quality of life.

The introduction of biologics into the therapeutic arsenal enabled a notable improvement of the clinical outcome of patients affected by skin psoriasis as well as psoriatic and rheumatoid arthritis. However, the efficacy of the currently available agents varies from patient to patient, and a still considerably high number of subjects fail to respond. Since the suboptimal response and the lack of prognostic predictors constitute significant health and economic burden, further research directed towards identifying novel therapeutic targets for the treatment of psoriasis/PsA and RA is needed.

Interestingly, the specific targeting of IL-36 by blocking its receptor (IL-36R) has already shown compelling anti-inflammatory effects in skin psoriasis; however, exploiting the properties of the antagonists IL-37 and IL-38 may represent an even more powerful weapon to inhibit IL-1-, Toll-Like Receptor (TLR)-, and IL-36-driven inflammation. Here, we will provide a comprehensive and updated description of the molecular and biologic features of the IL-36, IL-37, and IL-38 agonists and antagonists, particularly in the context of psoriasis, PsA and RA. At the same time, we will explore the therapeutic potential of targeting the IL-36 axis to control skin and joint inflammation.

## 2. IL-36, IL-37, and IL-38: A Complex Group of Pro- and Anti-Inflammatory Cytokines

Even if the cytokines of the IL-1 family share common maturation and signaling pathways, each cytokine retains peculiar properties and specific mechanisms of action. Indeed, except for IL-1RA, all the cytokines of the IL-1 family do not present a signal peptide and are not secreted via the classical endoplasmic reticulum or Golgi apparatus. In this section, we will describe common and specific aspects of the maturation, secretion and activation of IL-36, IL-37, and IL-38, and briefly mention their signaling processes, which have been recently thoroughly reviewed by Bassoy and colleagues [7].

### 2.1. Maturation

Although IL-1α and β bind a common receptor and have a similar effect, they do not require the same processing to become fully active. While the IL-1α precursor is already active and works as an alarmin in the tissue, the IL-1β precursor needs to be processed to become fully functional. Caspase-1 has been demonstrated to be the primary enzyme responsible for IL-1 maturation [8]. Caspase-1 itself must be activated by the inflammasome, a cytoplasmic multiprotein complex activated by diverse Pathogen-Associated Molecular Patterns (PAMPs) or Damage-Associated Molecular Patterns (DAMPs). Members of the IL-1 family have a consensus sequence that plays an essential role in maintaining their three-dimensional structure; this sequence is composed of three amino acids (aa) A-X-D, where A is an aliphatic aa, X can be any aa, and D is an aspartic aa that does not belong to the specific caspase-1 recognition sequence. The N-terminus of the fully active protein is usually placed nine aa before this sequence, allowing the formation of the first beta sheet structure, a hallmark of the IL-1 family [9].

#### 2.1.1. IL-36 and IL-36Ra

IL-36α, β, γ, and IL-36Ra cytokines, similarly to IL-1β, need to be processed to acquire their full agonist or antagonist activity. In its native form, IL-36Ra has no antagonist ability; similarly, IL-36α, β, and γ are 100–1000 times less active than their processed counterparts [10]. The A-X-D sequence rule has also been confirmed for IL-36α, β, γ, and IL-36Ra by Towne and colleagues in 2011 [10]. Recently, neutrophils proteases have been identified as the chief regulators of the processing of all the IL-36 family members, although with different specificity and affinity. Neutrophil elastase (NE) is the key enzyme required for enhancing IL-36Ra activity, especially in the context of psoriatic skin inflammation [11]. IL-36α seems to be activated by both NE and cathepsin-G, however, with differential patterns. Conversely, whereas cathepsin-G and proteinase-3 preferentially activate IL-36β, IL-36γ can be processed by NE, proteinase-3, cathepsin-G [12], and cathepsin-S [13], the last being particularly important for enabling the IL-36γ-related inflammation in skin psoriasis [14]. Neutrophil extracellular traps (NETs), in addition to their antimicrobial role, can also serve as a platform for the activation of IL-1α and IL-36 cytokines through NETs-associated cathepsin-G and NE [15]. Thus, neutrophils appear to be the principal cells responsible for IL-36 cytokines maturation via their secretory mechanisms. Therefore, they play an essential regulatory role on the IL-36-axis activity in skin- and joint-related inflammatory diseases [16].

#### 2.1.2. IL-37

The six exons of the *IL-37* gene encode five isoforms (IL-37a, IL-37b, IL-37c, IL-37d, and IL-37e), of which IL-37b is the best characterized so far. Similar to other members of the IL-1 family, the N-terminus of IL-37 does not contain a signal peptide but encloses a caspase-1 cleavage site, which only partially mediates the maturation and anti-inflammatory activity of IL-37 [17,18]. However, in the presence of caspase-1 mutations, the IL-37 ability to translocate into the nucleus and form a molecular complex with Smad3 to downregulate the transcription of specific key genes [19] is impaired [17]. Additional in silico-predicted IL-37 cleavage sites were described by Ellidson and colleagues in 2017 and included Cathepsin K, elastase-2, and matrix metalloproteinase (MMP)-9 cleavage sites [20]. Further studies will help to understand the maturation processes of all the IL-37 isoforms fully.

#### 2.1.3. IL-38

As described for the other members of the IL-1 family, likewise, IL-38 is released from cells independently of the presence of a signal peptide. The IL-38 maturation process has yet to be completely unraveled. Interestingly, Mora and colleagues discovered that, in vitro, IL-38 is N-terminally processed under apoptotic conditions [21], but they failed to identify the exact breakdown site and the enzymes responsible for the protein cleavage. The hypothetic maturation processes enabling the activation of IL-38 were extensively reviewed by Garraud and colleagues in 2018 [22]. The degree of maturation is particularly crucial for IL-38 since it can have antithetic effects on macrophages depending on its size. Indeed, while the full length IL-38 is able to increase the IL-6 production, the cleaved form downregulates IL-6 expression by binding IL-1 receptor accessory protein-like 1 (IL-1RAPL1) and, subsequently, inhibiting the Jun N-terminal kinase (JNK) pathway [21]. Discovering the details of the IL-38 maturation process will be essential to understand and exploit its impact on regulating inflammatory processes.

### 2.2. Receptors and Intracellular Signaling

**Figure 1:**
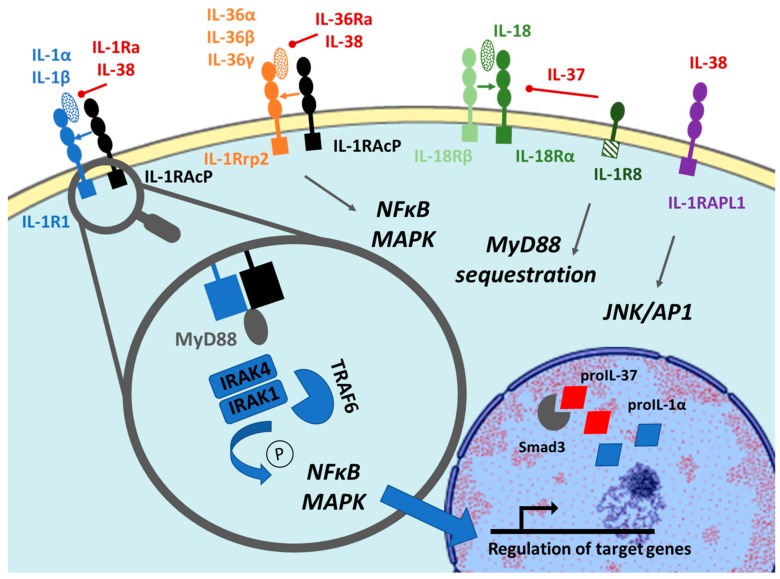
Overview of the IL-1, IL-36, IL-37, and IL-38 receptors and intracellular signaling. IL-1β binds the IL-1R1 receptor. Activated IL-1R1 recruits the IL-1RAcP common subunit and enables MyD88 to form a complex of signalization with IRAK and TRAF, which leads in turn to the phosphorylation/activation of the downstream signaling. A counter-regulatory pathway is represented by IL-1β binding its decoy receptor IL-1R2 (membrane-bound or soluble) (not shown). IL-36α, β, and γ bind to the IL-1Rrp2 and recruit the common subunit IL-1RAcP, inducing similar downstream signaling cascades as IL-1β. IL-37 binds IL-18Rα, which recruits IL-1R8 instead of the usual IL-18Rβ, causing sequestration of Myd88 and transduction of a weak signal because of the IL-1R8 mutated intracellular domain. IL-38 might be able to bind IL-1R1, IL-1Rrp2 and/or IL-1RAPL1 but further studies need to confirm its preferential intracellular mechanism of action. IL1R = IL-1 Receptor; IL-1RAcp = IL-1 Receptor Accessory Protein; IL-1RAPL1 = IL-1 Receptor Accessory Protein Like 1; IL-1Rrp2 (or IL-1RL2) = IL-1 Receptor Like 2; MyD88 = Myeloid Differentiation Primary Response Protein 88; IRAK = IL-1R-Associated Kinase; TRAF = TNF Receptor-Associated Factor; JNK = Jun N-terminal Kinase; AP1 = Activator Protein 1; NFκB = Nuclear Factor-kappa B; MAPK = Mitogen-Activated Protein Kinases.

The common structure of the receptors of the IL-1 family cytokines is characterized by three extracellular Ig domains and an intracellular Toll/IL-1 Receptor (TIR) domain. Similarly to TLR, the IL-1 family receptor can recruit the adaptor protein myeloid differentiation primary response protein 88 (MyD88) following the dimerization into a complex of signalization. So far, four distinct complexes have been described: IL-1R (IL-1R1 and IL-1 receptor accessory protein (IL-1RAcP)), IL-33R (ST2 and IL-1RAcP), IL-18R (IL-18Rα and β), and IL-36R (IL-1 receptor like 2 (IL-1RL2) or IL-1Rrp2- and IL-1RAcP). IL-1R2 and IL-18 binding protein (BP) lack the intracellular domain and act as decoy receptors by competitively linking to IL-1β and IL-18, respectively, and preventing their binding to IL-1R1 and IL-18R. The recently discovered TIR8 (also known as IL-1R8) is another exception to the characteristic structure as it contains one extracellular domain and one mutated TIR intracellular domain. This particular domain competes, in a decoy fashion, with the activated IL-1R or TLR complex, eventually leading to a decreased intracellular signaling [23]. Figure 1 summarizes and graphically represents the receptors and intracellular pathways activated by IL-1, IL-36, IL-37, and IL-38.

#### 2.2.1. IL-36 and IL-36Ra

IL-36α, β, and γ bind their cognate receptor IL-1Rrp2 and mediate the recruitment of the common chain IL-1RAcP. The subsequent dimerization of the cytosolic TIR domains and the recruitment of post-receptor signal transducers lead to the formation of a complex signalosome. This activates mitogen-activated protein kinases (MAPK) and nuclear factor-kappa B (NFκB) pathways [24,25] via several mediators, such as the IL-1R-associated kinases (IRAK) (1 and 4) and the signaling adaptor TNF receptor-associated factor 6 (TRAF6).

Overall, the activation of IL-36-dependent downstream signaling pathways induces the upregulation of proinflammatory genes including *IL-8* or *IL-6*. The A471T polymorphism of the IL1Rrp2 TIR domain, occurring in 2% of the population, leads to a reduced IL-36R signalization by diminishing the interaction between the two elements of the receptor (IL-1Rrp2 and IL-1RAcP) [26]. IL-36Ra specifically antagonizes the proinflammatory activity of the three IL-36 cytokines by binding IL-1Rrp2 with a higher affinity than the agonists [27]. IL-36Ra subsequently prevents the recruitment of the common subunit IL-1RAcP, mirroring the same inhibitory mechanism used by IL-1Ra for antagonizing IL-1α and β.

#### 2.2.2. IL-37

In 2002, for the first time, it had been proposed that IL-37 could bind IL-18Rα [18] with low affinity and, in turn, antagonize the effects mediated by this receptor. At the same time, a different group demonstrated that IL-37 could also bind IL-18BP and recruit IL-18Rβ to prevent the formation of the active complex IL-18R [28]. In addition to these possible mechanisms of action, further studies confirmed that IL-37 is a nonclassical inhibitor that can bind IL-18Rα and subsequently favor the recruitment of IL-1R8 instead of the usual IL-18Rβ [29]. IL-1R8 TIR domain lacks the Ser447 and Tyr536 residues; therefore, its binding to MyD88 results in poor signal transduction and triggers multiple intracellular switches that block the inflammation [29] and regulate inflammatory and immune processes in various pathologic conditions [30]. Noteworthy, at high concentration, IL-37b tends to form homodimers, which limit its bioactivity by either reducing the affinity for IL-18Rα or restricting the recruitment of IL-1R8. IL-37 homodimerization represents a possible autoregulatory mechanism to hinder further immunosuppression [31].

As mentioned above (2.1.2), and similarly to IL-1α or IL-33, IL-37 is also a “dual function” cytokine able to translocate into the nucleus and bind nuclear DNA to exert regulatory functions on gene transcription. This process is caspase-1-dependent and relies on the formation of a Smad3-IL-37 complex in the perinuclear space, followed by its subsequent translocation into the nucleus. The role of Smad3 is demonstrated by the lost ability of IL-37 to suppress cytokine-induced inflammation in response to Smad3 inhibition [19].

#### 2.2.3. IL-38

As noticed above concerning the maturation process of IL-38, little is also known about its exact molecular mechanism of action. Since 2001, several hypotheses, occasionally contradictory, have been postulated. IL-1R1 was initially proposed to be an IL-38 receptor [32]; however, this has not been consistently confirmed later on. Van de Veerdonk and colleagues suggested that IL-38 could instead bind IL-1Rrp2, as IL-36Ra does [33], and mediate a 42% reduction of the IL-36-dependent IL-8 production by human peripheral blood mononuclear cells (PBMC) (in comparison with the 75% IL-36Ra-mediated). Interestingly, IL-38 seems to work as a nonclassical inhibitor, being more active at low (10 ng/mL) rather than high doses (1 μg/mL) [33]. A recent study proposed that IL-38 might also bind IL-1RAPL1, and confirmed the role of IL-1R1, but not IL-1Rrp2, as an additional IL-38 cognate receptor [21].

Overall, since conflicting data exist on IL-38 binding partners further studies are needed to delineate its specific molecular mechanism.

## 3. Expression and Role of IL-36 Cytokines in Inflamed Skin and Joints

Although IL-36 cytokines have long been considered “less powerful” counterparts of IL-1β, their critical role in inflammatory conditions, such as psoriasis, is now well recognized. In this section, we will describe the expression and functions of the IL-36 family members within the tissues and organs primarily involved in psoriasis: PsA and RA.

### 3.1. Expression and Role in The inflamed Skin

IL-36 and IL-36Ra are physiologically present in the skin, but their expression is enhanced in psoriasis [34]. They are mainly produced by keratinocytes but also by macrophages or dendritic cells [35,36], and their release is upregulated in vitro by various stimuli like proinflammatory cytokines or TLR agonists (e.g., lipopolysaccharide (LPS), double-stranded (ds) RNA) [37,38,39,40]. In both human and murine psoriatic skin samples IL-36α, β, γ, IL-36Ra, and IL-38 are constitutively detectable but only IL-36α, γ, and IL-36Ra are selectively further induced during active inflammation [40]. IL-36 cytokines are also upregulated in anti-TNF-induced psoriasiform lesions in patients with Crohn’s disease [41], and might be involved in the pathogenesis of allergic dermatitis [42], alopecia [43] and Kindler syndrome [44].

Psoriatic skin lesions are characterized by hyperproliferation and altered differentiation of keratinocytes. Consequently, the release of proinflammatory mediators acting on immune cells sustains a self-amplifying loop able to perpetuate the cutaneous inflammatory process [45].

In this context, IL-36 cytokines, in cooperation with IL-17, negatively regulate keratinocytes differentiation and induce their proinflammatory phenotype [46,47] and the development of skin lesions. IL-36 plays an important role also directly on myeloid immune cells. Upon IL-36 stimulation, dendritic cells overexpress specific activation markers such as cluster of differentiation (CD)80, CD86, or class I major histocompatibility complex (MHC) and produce IL-1β, IL-12, IL-23, IL-6, and TNFα but also chemokine (C-C motif) ligand 1 (CCL1), chemokine (C-X-C motif) ligand 1 (CXCL1), and granulocyte-macrophage colony-stimulating factor (GM-CSF) in an IL-36R-dependent manner [48,49]. Langerhans cells and M2 macrophages (i.e., alternatively-induced macrophages) are similarly prompted by IL-36 to increase their proinflammatory activity [50]. IL-36R is expressed by CD4/CD8 T cells and B cells. Unlike other receptors of the IL-1 family that are mostly detected on polarized T cells (e.g., IL-1R), IL-36R is primarily found on the surface of naïve CD4 T lymphocytes, suggesting that IL-36 plays a role in initiating the immune response. The ability of IL-36 to regulate T cells biologic activity is of fundamental importance in psoriasis since this disease has been traditionally considered to have a T cell-mediated pathogenesis [51]. Several studies have demonstrated that IL-36 is involved in the maturation of T cells by increasing CD4 T cells proliferation [52], inducing T helper (Th) 1 polarization of Th0 cells [53], and directly triggering IL-17 production by murine CD4 T cells [35].

IL-36γ is also able to activate endothelial cells and promote leukocyte recruitment during skin inflammation through the induction of the expression of adhesion molecules vascular cell adhesion protein (VCAM)-1 and intercellular adhesion molecule (ICAM)-1 [54].

Concerning the regulation of the IL-36 family members by other cytokines, it seems that IL-22 and the Th17-related cytokines enhance the IL-36 expression within psoriatic lesions in murine models and human tissues [37], thus sustaining an autocrine and paracrine amplification loop between IL-36 and Th17 cytokines [37,47]. Interestingly, the anti-inflammatory effects of corticosteroids (e.g., dexamethasone) in mouse psoriatic skin are also driven by the disruption of the positive feedback loop between IL-36 and the Th17 axis [55]. Furthermore, the IL-36Ra deficiency exacerbates psoriasis in animal models [35] and the Deficiency of the interleukin-36 receptor antagonist (DITRA) is a recognized human syndrome characterized by generalized pustular psoriasis [56]. Some genetic polymorphisms in the IL-36β locus are also known to be associated with a higher susceptibility to psoriatic arthritis [57]. In mice, IL-36α is essential for the development of psoriasis. In fact, transgenic mice overexpressing IL-36α have severe skin inflammation, which is further intensified by IL-36Ra deletion [58]. Vice versa, IL-36α deficiency significantly reduces skin lesions development, while the specific deletion of IL-36β or γ does not affect the severity of the disease [59]. Mice lacking both IL-1R1 and IL-36α are disease-free in imiquimod-induced skin inflammation [60]. IL-36 cytokines have also been shown to be involved in prompting neutrophil infiltration and pustules formation in psoriatic lesions [61].

In keeping with the critical role played by IL-36 in driving psoriatic-like skin inflammation and after the success of preclinical studies [60], IL-36R blocking antibodies have been developed for the treatment of psoriasis and are currently being tested in clinical trials [62,63].

### 3.2. Expression and Role in the Inflamed Joints

In the synovial tissue of RA patients, IL-36 cytokines are expressed by various cells: plasma cells, macrophages and, to a lower extent, fibroblasts, endothelial cells, and dendritic cells [40,64]. During the course of collagen-induced arthritis (CIA) in mice, the gene expression of IL-36α, -β, and -γ, and IL-36Ra is locally enhanced in the joints at the peak of inflammation [40]. Fibroblast-like synoviocytes (FLS) from RA synovial membranes express IL-36R, and, upon IL-36 stimulation, they proliferate and produce proinflammatory cytokines, chemokines, and MMPs [65,66]. Besides, IL-36 cytokines have recently been demonstrated to mediate the cross-talk between plasma cells and FLS within the inflamed joints, eventually supporting the maintenance of autoreactive B cell niches [66].

Approximately 40% of subjects with RA are characterized by synovial ectopic lymphoid structures (ELS) surrounded by plasma cells, which locally produce pathogenic autoantibodies and may influence the chronicity of the inflammatory response [67]. Interestingly, IL-36 is also associated with the presence of ELS in other organs and diseases, e.g., colorectal cancer [68]. A role for IL-36 in inducing and maintaining ELS formation in RA synovium is therefore plausible and potentially exploitable for therapeutic purposes.

Furthermore, IL-36γ can promote T cell differentiation towards IL-9-producing lymphocytes (Th9) [69], which have been associated with augmented neutrophils survival and enhanced Th17 differentiation in the synovial tissue [70]. Not surprisingly, Th9 cells are enriched in peripheral blood and synovium of patients with inflammatory arthritis [71,72], and their presence correlates with disease activity in RA [70].

Although IL-36 cytokines are widely expressed within the synovial tissue, they seem to be generally dispensable for driving local and systemic inflammation in autoimmune arthritis. The silencing of IL-36R with blocking antibodies or by inhibiting its gene expression does not affect inflammation and bone destruction in several experimental models of arthritis [73,74,75], differently from that observed in models of psoriasis. However, it has been described that a subset of RA patients (~20%) is characterized by an elevated agonists (IL-36α, β, and γ)/antagonists (IL-36Ra and IL-38) ratio, as found in more than 90% of patients with psoriasis [40].

The presence of different subgroups of RA patients could be explained by the considerable heterogeneity of the human synovial tissue during inflammatory joint diseases [76]. It is plausible, therefore, that the subpopulation of RA patients with an increased IL-36 agonists/antagonists ratio has a peculiar synovial histological pathotype and/or a specific clinical phenotype. Since the histological and clinical features of this group of patients who could potentially benefit from IL-36 inhibition have not been defined yet, further research in this field will be critically important, particularly in keeping with the still significant rate of non-responders to the currently available treatment.

In line with the importance of IL-36 cytokines in driving skin psoriasis and the recognized role played by Th17-related cytokines in the development of PsA [5], we hypothesized that the IL-36 axis could be actively involved in driving synovial inflammation in PsA. The available knowledge on this topic is relatively little and, so far, a single study has confirmed the expression of IL-36α in PsA synovium [64]. Our data (unpublished, manuscript in preparation) reinforce this concept but also further suggest that the impaired balance between IL-36 agonists and antagonists contributes to the persistent inflammatory response that characterizes the inflamed synovium.

**Figure 2:**
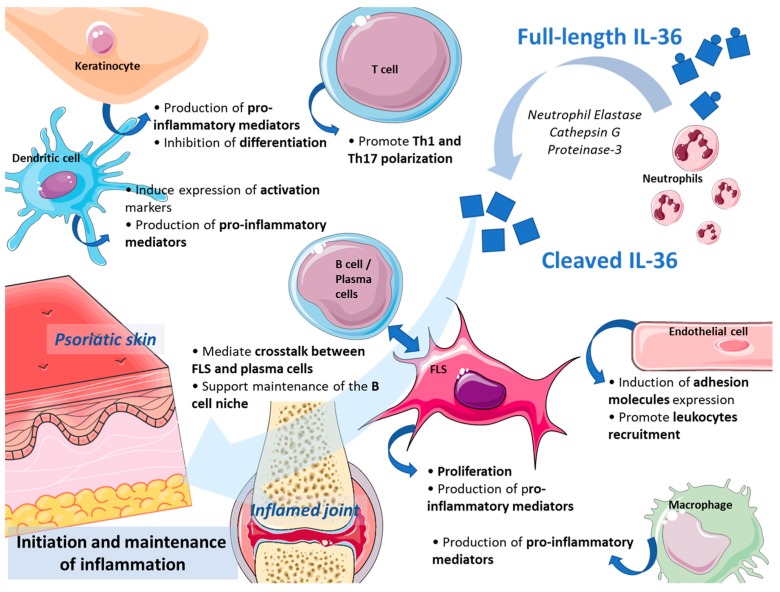
IL-36 agonists drive skin and joint inflammation. IL-36 cytokines are cleaved and activated by neutrophil proteases and act on multiple immune and resident stromal cells through their specific receptor complex IL-36R to initiate and amplify the inflammatory cascade. FLS = fibroblast-like synoviocytes; Th = T helper.

The main functions exerted by IL-36 agonists on inflammatory cells involved in skin and joint inflammation are represented in Figure 2.

### 3.3. Are IL-36 Cytokines a Good Target in Skin- and Joint-Related Inflammation?

Biologic agents have been introduced about two decades ago and have since then carried a significant improvement in the clinical outcome of patients affected by a range of diseases, such as psoriasis, PsA and RA. Biologics include drugs targeting proinflammatory molecules and pathways (e.g., TNF, IL-6, or the IL-17/IL-23 axis), specific population of cells (e.g., B cells) or immune mechanisms (e.g., T cell costimulation). However, up to 30 to 40% of the treated patients do not respond adequately to the available agents, either immediately after the initial treatment (primary failure) or by losing efficacy over time (secondary failure). The sequential use of the available molecules is currently based on “trial-and-error” but a stratified and personalized approach would certainly increase the rate of responders and reduce both the exposure to side effects and the economic impact related to the use of inappropriate drugs [77].

Importantly, different organ-dependent (i.e., skin versus joints) rates of response have been observed in PsA and thoroughly discussed in a recent review published by our group [5]. Moreover, distinct pathogenic mechanisms driving the development of skin or synovial inflammation must be considered when assessing the divergent efficacy of the biologics in PsA.

A better characterization of the pathways contributing to the chronic inflammation in psoriasis, PsA and RA would facilitate the development of new effective drugs. Here, we discuss the potentiality of targeting IL-36 in both skin- and joint-related inflammation.

It is now well accepted that IL-36 cytokines are paramount in psoriasis pathogenesis, and IL-36 receptor inhibition represents a promising therapeutic strategy for treating generalized pustular psoriasis (GPP) and palmoplantar pustulosis (PPP). Two phase I trials are currently evaluating the safety and pharmacokinetics of two different IL-36R blocking antibodies (ANB019 and BI655130) [78,79]. In the attempt to develop additional strategies to target the activation of the IL-36 cytokines, small molecules inhibiting the elastase have been generated. Since their efficacy to reduce IL-36 activation has been proven [80], they might represent a novel approach to inhibit IL-36-driven inflammation in psoriasis and other IL-36-dependent inflammatory diseases [81].

Whether or not the same pathogenic mechanisms sustain psoriasis and PsA is still under debate. Nevertheless, the significant neutrophil component characterizing the PsA synovium [82] and the well-known relevance of the Th17-related cytokines in the PsA development suggest that the IL-36 axis is a potential new therapeutic target in PsA with synovial inflammation (manuscript in preparation).

Several preclinical studies failed to demonstrate a pivotal role of the IL-36 cytokines in driving synovial inflammation in RA. However, analysis of human rheumatoid synovial tissue emphasized that at least a subset of patients defined by a high IL-36 agonists/antagonists ratio, similar to the lesional skin in psoriasis, might benefit from the inhibition of the IL-36 pathway [40].

Even if preliminary data on the effectiveness of IL-36 neutralization are encouraging, combined inhibition of the IL-1/IL-36 axis would be even more efficient and, possibly, necessary for obtaining a meaningful clinical effect.

We learned indeed that the single inhibition of IL-1α/β was of limited efficacy in treating RA patients, despite extremely promising preclinical data [83]; likely, this discrepancy relates to the redundancy of other proinflammatory compensatory pathways such as TNFα, IL-6, or other IL-1 family members like IL-36. In this context, the two newly discovered cytokines IL-37 and IL-38 might play a revolutionary role thanks to their ability to broadly inhibit the IL-1 and the TLR-mediated inflammation.

In the next section, we will review the therapeutic potential of IL-37 and IL-38 in the skin and joint inflammation.

## 4. IL-37 and IL-38, Broad Inhibitors of Skin and Joint Inflammation

### 4.1. Anti-Inflammatory Role of IL-37 in Skin and Joints

Although IL-37 is not a direct inhibitor of IL-36 cytokines, Nold-Petry and colleagues demonstrated that, through its binding to IL-1R8, IL-37 could limit TLR-, IL-1-, IL-18-, IL-33-, and IL-36-mediated inflammation [29]. Indeed, IL-1R8 interacts with and usurps molecules such as IRAK or TRAF6, involved in the downstream signaling of TLR and IL-1 family member cytokines, eventually limiting the activation of the signal. Not surprisingly, IL-1R8 deficient mice display a hyperinflammatory phenotype, are more susceptible to psoriasis, and develop more severe arthritis [84].

The critical role of IL-37 in inhibiting skin inflammation has been identified and described in human and animal models of psoriasis. IL-37 is less expressed in psoriatic skin lesions compared with non lesional skin [85,86], and can downregulate the production of key mediators like IL-8, IL-6, or S100 calcium-binding protein A7 (S100A7) involved in the development of psoriasis in mice [87]. These results suggest that the exogenous replenishment of IL-37 represents a promising therapeutic strategy for patients with psoriasis.

Although data in this field are somewhat limited, IL-37 seems to be also involved in preventing joint inflammation. Intra-articular injections of recombinant (rh) IL-37 or adenovirus encoding human IL-37 in mice with collagen-induced [88] or streptococcal cell wall (SCW)-induced arthritis [89] drive the downregulation of locally produced IL-17 and other Th17-related cytokines and ameliorate arthritic symptoms.

Furthermore, Tang and colleagues recently discovered that IL-37 could inhibit osteoclastogenesis [90]; this ability is particularly relevant for its potential therapeutic use in RA. Indeed, the typical bone erosions observed in RA patients are driven by the activation of osteoclasts and represent a major cause of pain and disability [91]. Somehow, unexpectedly, IL-37 levels in plasma and PBMCs in patients with RA are significantly higher compared with healthy controls [88] and correlate with the presence of activated T cells and the disease activity [92]. Conversely, the level of expression of IL-37 within the synovium of RA patients is not dissimilar from healthy controls [89].

**Figure 3:**
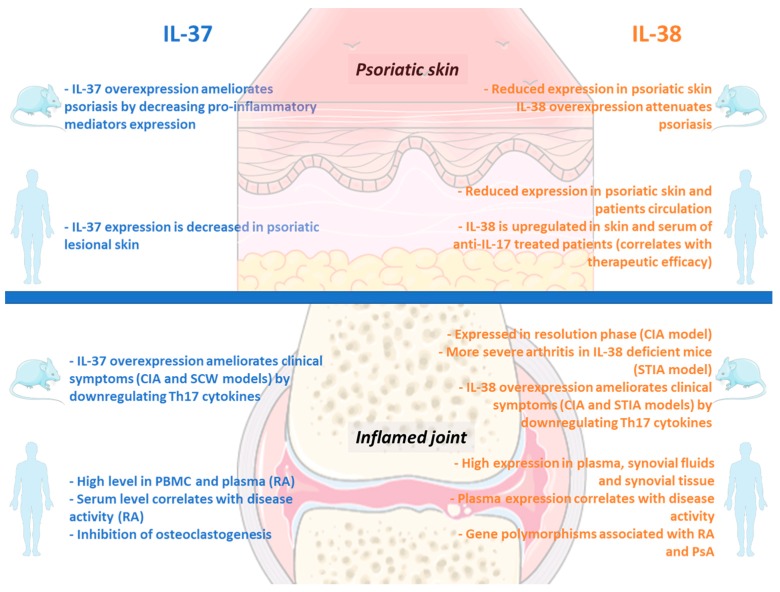
Main activities of IL-37 and IL-38 on skin and joints inflammation in mice and human. CIA = Collagen-Induced Arthritis; SCW = Streptococcal Cell Wall; PBMC = Peripheral Blood Mononuclear cells; RA = Rheumatoid Arthritis; STIA = Serum Transfer Induced Arthritis; Th = T helper; PsA = Psoriatic Arthritis.

Figure 3 (left panel) summarizes the primary roles and functions of IL-37 in human and mice skin and joints.

Even if further studies are needed to clarify the divergent expression of IL-37 in the circulation and within the synovium, overall, these data suggest that IL-37 might be exploited not only to treat psoriasis but also RA. To date, nothing is known about the direct role of IL-37 in PsA pathophysiology. The lower circulating levels of IL-1R8 observed in PsA patients might suggest a protective role [93], but additional studies are called to clarify these aspects.

### 4.2. Anti-Inflammatory Role of IL-38 in Skin and Joints

The *IL-38* gene expression profile in skin and joints is the opposite of the IL-36 agonists and IL-36Ra, with IL-38 mRNA being significantly reduced in the inflamed skin [40]. In addition, during the course of CIA in mice, IL-38 articular expression is increased lately in the resolution phase of inflammation in comparison with IL-36/IL-36Ra that are induced at the peak [40]. It is plausible to hypothesize that the lack of IL-38 may contribute to the persistent chronic inflammatory response characterizing psoriasis, RA, or PsA.

Alike IL-37, IL-38 has been shown to globally reduce the IL-1-, IL-36-, and TLR-mediated inflammation but with different mechanisms. IL-38 can act “directly” on the IL-36 axis by binding IL-36R (as IL-36Ra). Instead, IL-37 cannot directly modulate the IL-36 cytokines but it exerts its effect via IL-1R8.

Thanks to the “direct” mechanism, IL-38 can decrease the IL-36-dependent IL-8 expression by human PBMCs [33] and inhibit the phosphorylation of MAPK/NFκB induced by IL-36γ in keratinocytes. In this way, IL-38 counteracts the proinflammatory activities played by IL-36 agonists on keratinocytes [94]. Consistently, the administration of IL-38 reduces the endogenous level of IL-36γ within the inflamed skin in mice [94].

The inhibitory action of IL-38 on the IL-36 pathway can also be ‘indirect’ through at least two additional mechanisms. On the one hand, since IL-36γ expression is enhanced by TLR4 activation, the IL-38-mediated inhibition of TLR signaling indirectly decreases the release of the agonist IL-36γ [39]. For instance, IL-38 reduces TLR4-mediated inflammation by significantly decreasing IL-6 and IL-23 produced by THP1 cells or primary M1 macrophages upon LPS stimulation [95,96]. Since blocking the TLR4 pathway in an animal model of DITRA syndrome significantly limits the auto-inflammatory response [97], the IL-38-mediated targeting of the TLR signaling in inflammatory skin conditions is encouraging.

On the other hand, the downregulation of Th17-associated mediators operated by IL-38 has rebound effects on IL-36, which is a potent inducer of IL-23 [54] and can feedback positively the loop with the Th17 cytokines. In fact, PBMCs treated in vitro with a combination of IL-38-siRNA and TLR-ligands produce more mediators involved in Th17 cells recruitment and activation (e.g., IL-6 or CCL2) [98].

The low endogenous levels of IL-38 [40,94] are not able to control the course of imiquimod-induced psoriasis in mice [99]; however, the exogenous administration of recombinant IL-38 attenuates the severity of the disease [94]. The supplementation of IL-38 is also able to improve the skin lesions in an animal model of systemic lupus erythematosus [100].

Concerning the effects of IL-38 on arthritis, the induction of serum-transfer induced arthritis (STIA) in IL-38-deficient mice determines a more severe phenotype and a higher joint expression of IL-1β and IL-6 compared with littermates control [101]. Accordingly, the overexpression of IL-38 in the joints of CIA and STIA mice can reduce Th17 cytokines production and improve the clinical scores of the disease [96].

As observed for IL-37, IL-38 expression in plasma, synovial fluid, and synovium of RA patients is higher in comparison with healthy or OA controls, and correlates with disease activity [40,102], thus suggesting its potential use as a diagnostic or prognostic biomarker in RA.

In psoriatic patients, instead, IL-38 levels are reduced in both plasma and affected skin [40,94], and the ratio between IL-36γ and IL-38 expression associates with disease activity. Interestingly, in patients treated with the anti-IL-17 agent secukinumab, IL-38 expression is upregulated and associates with the therapeutic efficacy [94]. Our unpublished data showed that IL-38 expression is significantly reduced in early treatment-naïve PsA patients in comparison with RA, suggesting that its exogenous replacement with therapeutic purposes is worth further research (manuscript in preparation).

Certain polymorphisms of the gene encoding *IL-38* have been found to be associated with RA, PsA, and ankylosing spondylitis, but also with cardiovascular diseases and C-reactive protein (CRP) levels, implying a broader inhibitory role of this molecule [57,103,104,105,106].

Altogether, the inhibitory activities of IL-38 on TLR, IL-1, and IL-36 pathways (represented in the right panel of Figure 3) will hopefully be tested and exploited for improving skin and joint inflammation in several diseases.

## 5. Conclusions

In this review, we focused on three autoimmune inflammatory diseases, namely Psoriasis, PsA and RA. Through a comprehensive revision of the up-to-date literature, we have thoroughly described the tissue-specific expression and roles of IL-36, IL-37, and IL-38.

A still significantly large group of patients with psoriasis, PsA or RA do not reach the remission status despite the notable improvement of the clinical outcome following the introduction of biologic agents such as TNFα blockers and those targeting the IL-23/IL-17 axis. Therefore, a better characterization of the pathways actively contributing to the chronic inflammation in these diseases will pave the way towards the discovery of novel therapeutics. Among the more promising targets, exploiting the IL-37/IL-38 pathways represents an innovative strategy for controlling the pathologic inflammatory response in several diseases.

## Abbreviations

IL	Interleukin
IL-1Ra	IL-1 Receptor antagonist
PsA	Psoriatic Arthritis
RA	Rheumatoid Arthritis
CASPAR	ClASsification criteria for Psoriatic ARthritis
IL-36R	IL-36 Receptor
TLR	Toll-Like Receptor
PAMP	Pathogen-Associated Molecular Pattern
DAMP	Damage-Associated Molecular Pattern
NE	Neutrophil Elastase
NET	Neutrophil Extracellular Trap
MMP	Matrix Metalloproteinase
JNK	Jun N-terminal Kinase
IL-1RAPL1	IL-1 Receptor Accessory Protein Like 1
TIR	Toll/IL-1 Receptor
MyD88	Myeloid Differentiation Primary Response Protein 88
IL-1RAcP	IL-1 Receptor Accessory Protein
IL-1RL2	IL-1 Receptor Like 2
BP	Binding Protein
MAPK	Mitogen-Activated Protein Kinases
NFκB	Nuclear Factor-kappa B
IRAK	IL-1R-Associated Kinase
TRAF	TNF Receptor-associated Factor
PBMC	Peripheral Blood Mononuclear Cells
AP1	Activator Protein 1
LPS	Lipopolysaccharides
ds	Doubled-stranded
CD	Cluster of differentiation
MHC	Major Histocompatibility Complex
CCL1	Chemokine (C-C motif) Ligand 1
CXCL1	Chemokine (C-X-C motif) Ligand 1
GM-CSF	Granulocyte-Macrophage Colony-Stimulating Factor
Th	T helper
VCAM	Vascular cell adhesion protein
ICAM	Intercellular Adhesion Molecule
DITRA	Deficiency of the Interleukin-36-Receptor Antagonist
CIA	Collagen-Induced Arthritis
FLS	Fibroblast-like Synoviocytes
ELS	Ectopic Lymphoid Structure
STAT	Signal Transducer and Activator of Transcription
GPP	Generalized Pustular Psoriasis
PPP	Palmoplantar pustulosis
S100A7	S100 calcium-binding protein A7
SCW	Streptococcal Cell Wall
STIA	Serum Transfer-Induced Arthritis
CRP	C-Reactive Protein
DMARD	Disease Modifying Anti-Rheumatic Drug
